# The crucial impact of NIH funding on allergy/immunology patient care and research

**DOI:** 10.1016/j.jacig.2025.100509

**Published:** 2025-06-03

**Authors:** Nora A. Barrett, Anna Nowak-Wegrzyn, Leonard B. Bacharier, Priya Bansal, Lynda G. Kabbash, Jordan S. Orange, Paul V. Williams, Frank S. Virant, Carla M. Davis, Rebecca Brandt, Thomas A. Fleisher, American Academy of Allergy, Asthma & Immunology Scientific Community Task Force for Allergy/Immunology Research

**Affiliations:** a Department of Medicine, Brigham and Women’s Hospital, Boston, Mass; b Department of Pediatrics, Hassenfeld Children's Hospital, New York University Grossman School of Medicine, New York, NY; c Department of Pediatrics, Monroe Carell Jr Children’s Hospital at Vanderbilt University Medical Center, Nashville, Tenn; d Division of Allergy and Immunology, Northwestern Feinberg School of Medicine, Chicago, Ill; e Division of Allergy and Inflammation, Beth Israel Deaconess Center, Boston, Mass; f Department of Pediatrics, Columbia University Vagelos College of Physicians and Surgeons, New York, NY; g Department of Pediatrics, Division of Allergy and Infectious Disease, University of Washington School of Medicine, Seattle, Wash; h Department of Pediatrics and Child Health, Howard University College of Medicine, Washington, DC; i American Academy of Allergy, Asthma & Immunology, Milwaukee, Wis

**Keywords:** National Institutes of Health

Through the innovative work of investigators supported by National Institutes of Health (NIH) funding, the United States is acknowledged as the global leader in biomedical research. This dominance has stretched over 80 years^1^ and has resulted in an unprecedented number of new therapies that have revolutionized medical practice. The NIH investment in the field of allergy and immunology has produced new biologic therapies and drugs that affect virtually all disorders cared for by allergists and immunologists, providing relief for patients experiencing the very common forms of allergic disease, including asthma, food allergy, and atopic dermatitis. Notably, *354 of 356 drugs approved by the US Food and Drug Administration over the 10-year period from 2010 to 2019 had their origins in NIH-funded research.* Moreover, ongoing NIH-funded research in immunology promises to treat, prevent, and even cure many more disorders. NIH funding has allowed physicians and investigators to advance the use of (1) gene therapy for severe combined immunodeficiency syndromes, hereditary angioedema, and alpha-1 antitrypsin deficiency; (2) kinase inhibitors for immune dysregulation and mastocytosis; (3) microbiome-based therapies for atopic dermatitis and ulcerative colitis; (4) complement inhibitors for hemolytic uremic syndrome, myasthenia gravis, and acute organ rejection; and (5) chimeric antigen receptor (CAR) T-cell therapies for an increasing number of cancers and autoimmune diseases, among many other treatments. Additionally, NIH-funded research is supporting major innovations in health care delivery, such as accelerating the integration of artificial intelligence into clinical medicine to reduce provider burnout, improving efficiency, and reducing health care costs.

The magnitude and pace of this pipeline is threatened due to a proposed 38% reduction in the NIH budget for fiscal year 2026 (Fig 1^2^). This reduction in US biomedical research has not been entertained over the past 8 decades, which have seen only gradually increasing investment and accelerating returns. Such a marked decrease in NIH funding will jeopardize the majority of phase 1 clinical trials and will drastically limit new drug development. Additionally, the proposal also challenges numerous economic metrics that are evidence for continued robust federal support for biomedical research, including:^1^^,^^3^

• Every dollar provided by the NIH results in approximately $2.50 returned to the local community in which the research is taking place;
• NIH funding provides more than 400,000 jobs at the supported medical centers located throughout America, including in every US state;
• NIH-supported institutions provide training of 29,000 individuals with MD degrees and between 8,000 and 10,000 individuals with PhD degrees annually; and
• Federally funded research yields approximately 1,100 US science-based startup companies annually.

**Fig 1:**
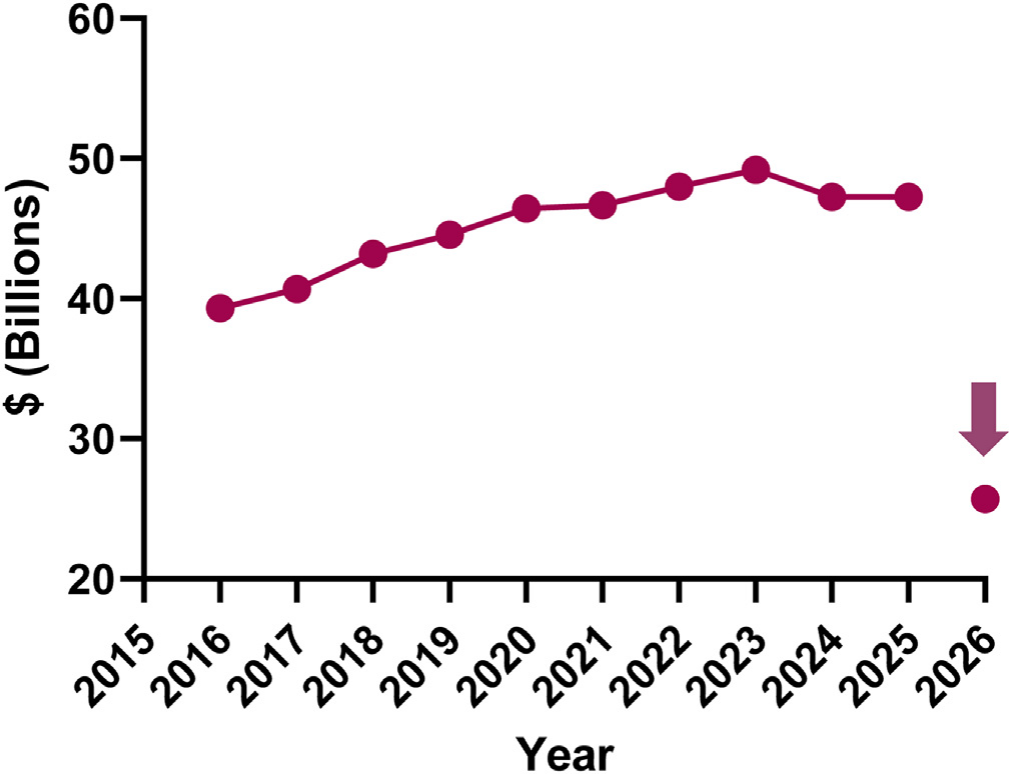
NIH annual budget, 2015-2025. ∗Inflation-adjusted, in fiscal year (FY) 2023 dollars, as per NIH funding: FY1996-FY2025 (2025).^2^*Pink arrow* indicates 2026 budget proposal.

Importantly, whereas inflation-adjusted costs of housing and goods have increased (reflected best in the Consumer Price Index) over the past 20 years, the rate of inflation-adjusted NIH funding in the fiscal year 2024 was –2.7% (and thus comparatively *less* than funding in 2003).^2^

The potential impact of major cuts in this highly productive public-private partnership that drives American leadership in biomedical research will have a lasting negative impact on patients, physicians, investigators, medical centers, and communities across America. The United States risks losing its leadership position in biomedical research, which will thwart new discovery at a time when new approaches to managing and even curing a host of devastating chronic disease are now within sight. As physicians and investigators involved in the management of patients with allergic and immunologic diseases, we must express our concerns that any significant cut in the NIH budget will have a catastrophic impact on the lives of our patients.

## Disclosure statement

Disclosure of potential conflict of interest: The following authors are supported by National Institutes of Health grants R01AI34989 (N.A.B.), U19AI095219 (N.A.B.), T32AI007306 (N.A.B.), U01AI170836 (A.N.-W.), U01AI181927 (L.B.B.), UG3OD035516 (L.B.B.), R01AI120989 (J.S.O.), R37 AI067946 (J.S.O.), R34AI157948 (C.M.D.).

## References

[1] Blank S. (2025). How the United States became a science superpower. Nature.

[2] National Institutes of Health (NIH), Funding: FY1996-FY2025. Congress.gov. https://www.congress.gov/crs-product/R43341.

[3] (2025). Association of American Medical Colleges.

